# Artificial Intelligence and the Discovery of Antibiotics: Reinventing with Opportunities, Challenges, and Clinical Translation

**DOI:** 10.3390/antibiotics15020233

**Published:** 2026-02-23

**Authors:** Bharat Kumar Reddy Sanapalli, Shrestha Palit, Ashwini Deshpande, Ramya Tokala, Dilep Kumar Sigalapalli, Vidyasrilekha Sanapalli

**Affiliations:** 1 Department of Pharmacology, School of Pharmacy and Technology Management, SVKM’s Narsee Monjee Institute of Management Studies (NMIMS), Deemed-to-University, Green Industrial Park, TSIIC, Jadcherla 509301, Hyderabad, India; bharathsanapalli@yahoo.in; 2 School of Pharmacy and Technology Management, SVKM’s Narsee Monjee Institute of Management Studies (NMIMS), Deemed-to-University, Green Industrial Park, TSIIC, Jadcherla 509301, Hyderabad, India; shresthapalit5@gmail.com; 3 Department of Pharmaceutics, School of Pharmacy and Technology Management, SVKM’s Narsee Monjee Institute of Management Studies (NMIMS), Deemed-to-University, Green Industrial Park, TSIIC, Jadcherla 509301, Hyderabad, India; ashwinideshpande4@gmail.com; 4 Athinoula A. Martinos Center for Biomedical Imaging, Department of Radiology, Massachusetts General Hospital and Harvard Medical School, Charlestown, MA 02129, USA; rtokala@mgh.harvard.edu; 5 Department of Biochemistry, University of Washington, Seattle, WA 98195, USA; 6 Department of Pharmaceutical Chemistry, School of Pharmacy and Technology Management, SVKM’s Narsee Monjee Institute of Management Studies (NMIMS), Deemed-to-University, Green Industrial Park, TSIIC, Jadcherla 509301, Hyderabad, India

**Keywords:** artificial intelligence, antimicrobial resistance, antibiotic discovery, machine learning, deep learning, generative models, antimicrobial peptides

## Abstract

Background: The outbreak and spreading of antimicrobial resistance (AMR) in a very short time has made most of the old-fashioned antibiotics ineffective, and thus new therapeutic substances have to be developed. The traditional methods of antibiotics discovery are defined by long periods of time, high levels of expenditure, and high rates of failure, which contributes to the necessity of new approaches. Artificial intelligence (AI) has become a disruptive technology that can be used to accelerate and optimize various steps of antibiotic discovery, such as target detection and virtual screening, new molecular design, and early-stage testing. Methods: This review provides an in-depth discussion of the role of AI methodologies in the form of machine learning, deep learning, natural language processing, and generative models in the discovery of small-molecule antibiotics and antimicrobial peptides (AMPs). The major areas that are discussed include virtual screening, pharmacokinetics optimization, resistance mechanism prediction, and AMPs design, which is accompanied by relevant case studies, including the AI-based discovery of Abaucin. Results: The article highlights how AI can be used in a synergistic relationship with synthetic biology, nanotechnology, and multi-omics data as a core component in the next generation of antimicrobial approaches, such as personalized therapy and predictive stewardship. The existing issues, i.e., the lack of data, bias in algorithms, and the translational divide between research and clinical use, are addressed, as well as suggested measures of responsible, collaborative, and ethical AI use. Conclusions: The combination of computational innovation with experimentation validation, AI-driven antibiotic discovery paves the way for a potent and scalable approach in addressing the rising threat of AMR.

## 1. Introduction

The current intensification of antimicrobial resistance (AMR) represents an intimidating menace to the health of the entire world, often described as one of the gravest dangers that modern medicine is facing. The growing number of drug-resistant infections, both the multidrug-resistant (MDR) and extensively drug-resistant (XDR), has limited the therapeutic options to common and severe infections related to the frequent ineffectiveness of many traditional antibiotics [1]. This crisis has been worsened by various reasons such as poor use and prescribing of current antibiotics, lack of proper stewardship, and lack of proper pipeline advancement of new antimicrobial agents. As a result, a deep and growing consumer health need has emerged to discover new antibiotics that would bypass existing resistance mechanisms and treat the resistant infections [2].

The classic antibiotic discovery processes have been known to be sluggish, expensive, and resource-heavy, and can also take more than ten years in the same process to identify a target and then get it through clinical approval. This long development process, together with high attrition rates and the difficulty of identifying compounds with acceptable pharmacodynamic and pharmacokinetic properties has led to the relative paucity of new antibiotics to be approved [3]. Additionally, the economic environment including low returns of investing in the development of antibiotics as compared to the chronic therapies has discouraged the pharmaceutical companies to invest highly in research on antimicrobials [4]. The growing health burden on the population is forcing the need to consider innovative approaches that can help overcome these limitations and accelerate the search and development of effective antibiotics. The increasing number of cases of AMR has come to a level that it has become an imperative to look for innovative ways to overcome these constraints and hasten the development stages of effective antibiotics.

**Figure 1:**
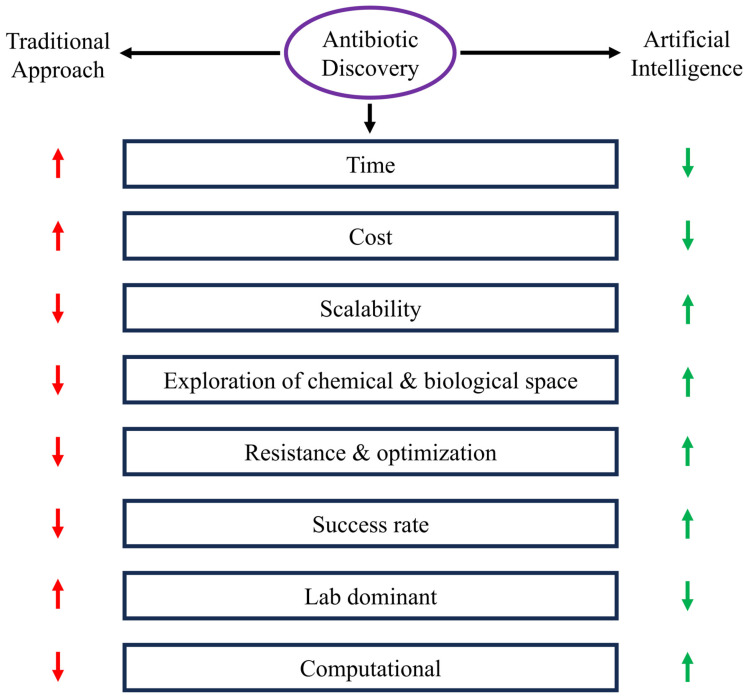
Relative comparison between the conventional methods of antibiotic discoveries and AI-based methods. The figure points out the most important parameters that affect the development of antibiotics, such as time, cost, scalability, chemical and biological space exploration, resistance management and optimization, success rate and reliance on laboratory workflow or computational workflow. Conventional methods are built around longer timescales, greater expenses, lack of scalability and intensive reliance on wet-lab testing, whereas AI-based methods are much quicker, cheaper, more scalable, and allow better exploration of chemical and biological space, are better at being optimized without resistance, and have higher overall success rates through computationally inspired decision-making.

In this regard, the use of computer-based approaches, particularly artificial intelligence (AI), has emerged as a particularly encouraging development which possesses the potential to affect a paradigm shift in the area of antibiotic development [5]. AI tools can be used to automate and improve various aspects of a drug development pipeline in antibiotics, ranging from screening to optimization to preclinical testing. With its ability to question the volume and complexity of data, analysis in AI might elucidate concealed trends, develop predictive models, and enable rational drug development [6]. AI is able to deal with the challenging scenario presented by resistant bacterial pathogens and can develop new approaches to revive the efficacy of antibacterial treatment (Figure 1). The present review summarizes recent developments in AI-based antibiotic discovery, including small molecules, antimicrobial peptides (AMPs), resistance prediction, and translational issues, to provide a coherent overview of how the data-driven approach is revamping the process of antimicrobial innovation.

### 1.1. Role and Potential of AI

The field of AI includes an entire set of computational methods with special focus on machine learning (ML), deep learning (DL), natural language processing (NLP), and reinforcement learning [7]. All of these approaches enable the machines to learn based on the obtained data and perform tasks that, in the past, required human intelligence. In the antibiotic discovery field, AI has transformed the traditional processes, such as literature mining, compound screening, and predicting drug properties, which are common processes in the field of antibiotic discovery and development [8]. Researchers are therefore in a better place to deal with the complex nature and size of drug-discovery pipelines using a novel methodological framework.

One of the key advantages of AI is that it has the ability to combine and analyze large data sets that include chemical properties, biological assay outcomes, genomic and pharmacokinetic measurements. The complex biological interactions can be modeled using AI algorithms and the molecular dynamics can be simulated, which makes the predictions based on the efficacy and toxicity more accurate compared to the traditional heuristic methods [9]. Such methodological developments help in the faster discovery of lead compounds with better activity profiles and safety margins to support previous go/no-go decisions in the development process.

Moreover, machine learning models and especially deep neural networks have demonstrated superiority in predicting molecular properties such as solubility, stability and binding affinity, which is critical in optimizing the antibiotic candidates. Combining AI with high-throughput experimental modalities and automated synthesis platforms has the potential to create entire pipelines in discovery enabling the rapid cycles of design thereby truncating the time lag between the conceptualization and clinical exploitation [10]. This synergy does not only enhance the speed with which the discovery is made but also provides cost efficiencies that cannot be ignored in the quest to revitalize the effort of antibiotic development.

### 1.2. Scope and Objectives of AI-Driven Antibiotic Discovery

The use of AI in the field of antibiotic discovery is a diverse field, including antibiotic peptides (AMPs), the discovery of small-molecule antibiotics, virtual screening, and mechanistic prediction. AMPs are a subclass of therapeutics with the ability to be broad-spectrum antimicrobial and slower to develop resistance and, therefore, make them a good target in AI-assisted design and optimization [11]. Simultaneously, AI-based discoveries are applied to small-molecule antibiotics, where molecular docking, pharmacokinetic modeling as well as molecular design are used as computational models.

The interdisciplinary cooperation is an essential aspect of long-term development of the AI-aided antibiotic research. Combining the knowledge of microbiology, computational biology, medicinal chemistry, data science, and clinical pharmacology promotes the creation of healthy algorithms with biological know-how [12]. The sharing of open data has become a critical element since it allows the combination of high-quality screening datasets necessary to train and validate predictive models, improving their generalizability and applicability.

**Figure 2:**
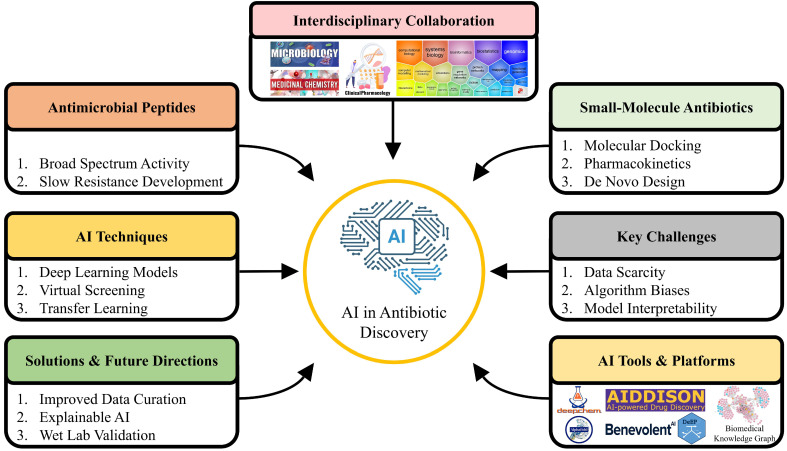
Theoretical demonstration of AI in the contemporary antibiotic discovery. The framework depicts the combination of antimicrobial peptides (AMPs) and small-molecule antibiotics with AI-based methods like DL, virtual screening, and transfer learning. The main issues such as the lack of data, algorithmic bias, and model interpretability are mentioned, and the future trends and solutions to them such as better data curation, explainable AI, and wet-lab validation are given. The figure also highlights the necessity of interdisciplinary teamwork in the areas of microbiology, medicinal chemistry, computational biology, pharmacology and systems biology with the aid of sophisticated AI tools and platforms to hasten and streamline the process of antibiotic discovery.

Although AI-based antibiotic discovery has promising prospects, it is faced with a number of challenges such as heterogeneity and scarcity of labeled datasets, algorithm biases, and model interpretability [13]. The future outlook focuses on the reduction in such issues by the introduction of better data curation practices, creation of explainable AI (XAI) systems and incorporation of wet-lab research to confirm computational forecasts. The potential of the strategic implementation of AI does not only lie in its ability to speed up the discovery but also in the ability to deliver personalized and adaptive therapeutic solutions to effectively fight AMR (Figure 2).

## 2. AI Technologies and Methodologies in Antibiotic Discovery

### 2.1. ML and DL Techniques

The field of AI application in antibiotics discovery is a wide field that includes the identification of AMPs, the discovery of small-molecule antibiotics, virtual screening, and mechanistic prediction. At the same time, AI-led discovery programs also consider small-molecule antibiotics, in which computational models are used in molecular docking, pharmacokinetic profiling, and de novo molecular design [14,15]. Representative platforms using these ML and DL methods to discover antibiotics are listed in Table 1.

The state-of-the-art DL architectures have increased the ability to perform tasks like ligand–receptor binding affinity prediction, de novo drug design, and resistance mechanism modeling significantly [16]. These models are useful in handling large-sized chemical libraries and thus enable virtual screening pipelines whereby high potential compounds are given priority out of millions of compounds in a collection. Additionally, transfer-learning and multi-task learning techniques confer the possibility to use common knowledge to solve similar problems to enhance the performance in data-sparse conditions, which is a frequent problem in antibiotic research. Such AI approaches are used to small-molecule antibiotics as well as AMPs, the biological aspects and therapeutic opportunities of which are discussed in subsequent sections.

### 2.2. Data Sources and Preprocessing for AI Models

The effectiveness of AI-based discoveries of antibiotics depends on the quality and diversity of the underlying data. Among the main data sources are chemical libraries with structural and functional annotations, multi-omics datasets which contain genomics, transcriptomics and proteomic studies of bacterial pathogens, and phenotypic screening datasets which determine the antimicrobial potency, and cytotoxicity [17].

Every one of these different datasets must be preprocessed by integration, normalization and standardization to ensure consistency and relevance. The issues of data quality, including stochastic noise, missing data, and class imbalance when the active compounds are not represented in comparison with inactive compounds are also formidable challenges. The reduction in such problems requires strict curative efforts, the implementation of data augmentation policies, and the implementation of algorithms that are resistant to unbalanced distributions [18].

The phenotypic data extracted from high-throughput screening platforms benefit through AI-based standardization and annotation tools, and, thus, have improved utility in model training. Synthetic Minority Over-Sampling Technique (SMOTE) and generative adversarial networks (GANs) techniques have been used to augment the thin set of positive samples [19].

Large-scale language models (e.g., BioGPT and ChemBERTa) have been used to enable the automatic identification of chemico-biological relationships, the generation of mechanistic theories, and the prediction of retrosynthetic routes that are outlined in the scientific literature. Such models can speed up the suggested candidate molecular architecture and reaction trajectories, alleviating the load of hypothesis testing on the antimicrobial research [20,21].

### 2.3. Generative Models and De Novo Drug Design

Generative AI models have become advanced tools to design new antibiotic candidates whose new molecular scaffolds are given desired pharmacological properties [22]. With the use of architectures like variational autoencoders (VAEs), GANs and reinforcement learning-based frameworks, such models can explore large chemical spaces that are larger than those covered by existing libraries of compounds [23].

Through such methodologies multi-objective optimization is enabled and allows the simultaneous optimization of parameters such as antimicrobial potency, cytotoxicity, synthetic feasibility and pharmacokinetic properties. One such example is the SyntheMol pipeline that uses Monte Carlo Tree Search algorithms, paired with graph neural networks, to create small-molecule antibiotics with a set of attributes optimized simultaneously [24]. In addition to small molecules, generative models have been applied to design AMPs, and they can search the enormous combinatorial sequence space to find peptides with high activity but avoid toxicity.

Generative AI is used to optimize the lead more quickly and discover chemically novel compounds through the refinements of model predictions in reaction to empirical data [25].

Molecular dynamic (MD) simulation is a computational method applied in the study of the dynamics of physical movements of atoms and molecules, using Newton equations, and solved numerically. It is used to determine the thermodynamic properties and the dynamics of complex systems by computing interatomic forces and following their evolution. With such a strategy, it is now possible to run modern MD simulations in scalable, cloud-based high-performance computing (HPC) and AI platforms [26], which can give the computational capacity required to conduct large-scale studies. At the same time, quantum machine learning (QML) currently has the center of interest, and its goal is to monitor complex quantum processes at the quantum–chemical interface between, e.g., antibiotics and their target, which may make ultrafast virtual screening possible [27].

### 2.4. Differentiating Bioinformatics Pipelines and AI Models in Antibiotic Discovery

The discovery of AI in antibiotics is not an isolated system but rather a component within a larger bioinformatics system. It is thus important to differentiate between the bioinformatics tools, which create and arrange biological information, and AI models, which learn the available information and make predictions and generate novel molecules.

Data generation, annotation and preprocessing are undertaken by bioinformatics pipelines [28]. These are genome sequencing and annotation websites, protein structure prediction applications (e.g., AlphaFold2 and RoseTTAFold), molecular docking systems (e.g., GNINA) and databases like PDB, ChEMBL, BindingDB, and AMP repositories. These tools encode raw biological or chemical data as structured machine-readable formats, like protein structures, molecular graphs, binding scores, and activity labels.

Based on these bioinformatics outputs, AI systems are operated. ML and DL models take bioinformatics-processed data as the training input to learn structure–activity relationships, predict antimicrobial activity, estimate ADMET properties, and produce new compounds [29]. As an example, docking tools replace protein structures predicted by AlphaFold2 with binding poses, which are then used as inputs for DL scoring functions or GNNs to predict binding affinity and selectivity. Recently, AlphaFold3 [30] has greatly expanded this capability by making it possible to directly predict protein small-molecule, protein-DNA, and protein-protein complexes. Compared to AlphaFold2, which is used to obtain only static protein structures, AlphaFold3 can be used to obtain biologically realistic target -ligand binding geometries enabling AI-assisted docking, GNN and DL scoring, which can use more accurate interaction geometries. The development enhances the accuracy of AI-based prediction of binding affinities, prioritization of hits, and optimization of antibiotic leads by structures against bacterial enzymes, membrane proteins, and resistance determinants [31].

Equally, in the discovery of AMPs, bioinformatics tools first extract peptide sequences in genomes or metagenomic databases and annotate them on the basis of physicochemical and evolutionary characteristics [32]. AI classifiers (including AMPs-Net), which learn discriminative patterns between active and inactive peptides and generative models (VAEs, GANs, transformers) that generate absolutely novel peptide sequences with optimized antimicrobial and toxicity properties, process these sequences [33].

Therefore, bioinformatics offers structured biological and chemical data, whereas AI offers learning, predictive, and generative intelligence. The workflow of antibiotic discovery is thus a closed-loop process: bioinformatics programs generate and annotate data → AI programs analyze and make hypotheses → experimental programs produce new data → the bioinformatics pipeline reprocesses the datasets → and the AI programs are retrained (Table 2). This division of labor provides scientific rigor, reproducibility and correct interpretation of AI-guided findings.

## 3. AI Applications in Small-Molecule Antibiotic Discovery

### 3.1. Virtual Screening and Molecular Docking Enhancements

AI technologies have significantly improved virtual screening methods, which are part of early-stage drug discovery, over the past several years. ML models make molecular docking simulations more accurate by providing better predictions for ligand–receptor interactions and thus reducing the number of candidate compounds to those which show high binding affinity to bacterial targets [34].

Using AI, QSAR (Quantitative Structure–Activity Relationship) models are able to rank compounds by antimicrobial activity very quickly, which then allows for more focused in vitro validation to be carried out. When docking is combined with AI-based ADME (Absorption, Distribution, Metabolism, and Excretion) and toxicity prediction processes, it becomes possible to identify candidates that have a drug-like profile with few liabilities and thus lower the risk of attrition [35]. In silico assessment of pharmacokinetic properties and interaction energies helps to facilitate the selection of agents eligible to be studied using expensive methods.

These kinds of wide-screening approaches have been exemplified by a study wherein the derivatives of natural products, specifically the curcuminoid compounds, were screened for the outer membrane protein targets of *Acinetobacter baumannii* (*A. baumannii*). In fact, the AI-assisted approaches developed by this study have been proven not just efficient but highly effective in detecting the lead compounds, which yielded favorable binding energy and pharmacokinetics, as validated by the results of the microbiological assays [36].

### 3.2. Optimization of Drug-Likeness and Pharmacokinetics

The optimization of drug-likeness factors has models at the center, which plays an extremely important role in the development of antibiotics since there are unusual conditions posed by bacterial membranes and host toxicity [37]. The models at a very early stage of development determine solubility, stability, and metabolites; hence, there are low chances of failures at a later stage.

DL and graph-based modeling are some of the approaches being used to explore the attributes of a molecule to enable the identification of potential adverse reactions and minimize the effect associated with off-target reactions [38]. These approaches enable the iterative development of the lead, and hence the feasibility and bioavailability of the drug are optimized. Furthermore, the AI system has the potential to predict both drug interactions and the potential resistance that might emerge in the future by modeling the interaction of the target and the nutrient metabolism of the bacteria.

All the above optimization steps, when combined in a computational–experimental cycle, are alleged to have the potential to dramatically lower the rates of attrition and quicken the movement of leads to preclinical stages of testing.

### 3.3. Case Studies: Successful AI-Identified Small-Molecule Antibiotics

Discovery of antibiotics based on AI has already yielded a number of clinically and biologically confirmed antimicrobial candidates. The summary of the representative AI-discovered antibiotics, their targets, and their computational strategies is presented in Table 3. These examples are discussed further in the following case studies by presenting the underlying datasets, AI models, and experimental validation procedures that resulted in the discovery of new small-molecule antibiotics which are effective against MDR bacterial pathogens [26].

#### 3.3.1. Halicin: Discovery of a New Broad-Spectrum Antibiotic by DL

A deep NN trained over a large set of chemical structures and experimentally measured antibacterial activity was used to discover Halicin. The model has been trained on intricate structure–activity relationships in addition to classical QSAR characteristics and applied to screen drug libraries containing in excess of 100 million molecules. The AI system focused on compounds that it was thought would inhibit the growth of bacteria and which was structurally distinct to the known antibiotics, which further enhanced the possibility of discovering molecules with new modes of action.

Halicin was one of the best candidates identified through this virtual screening. The activity was experimentally validated as being strong against several MDR- pathogens, such as *Acinetobacter baumannii*, *Mycobacterium tuberculosis*, and *Clostridioides difficile*. In vivo confirmation in murine infection models revealed that there was successful clearance of bacteria with minimal toxicity [39]. Mechanistic analyses found subsequently that Halicin interferes with the proton motive force of bacterial membranes, which is an alternative mechanism of action to the conventional antibiotics.

#### 3.3.2. Abaucin: ML-Guided Narrow-Spectrum Antibiotic Discovery

Abaucin was identified on a ML-trained virtual screening pipeline specifically trained on activity information against *A. baumannii*. By comparison, the AI model was tailored to detect compounds that specifically suppress this pathogen and minimize the chances of off-target effects on the useful microbiota, unlike broad-spectrum screens. The model filtered the thousands of compounds by combining the molecular descriptors, docking scores and predicted permeability across the bacterial outer membrane. The best molecules were tried in vitro, and Abaucin was identified as being highly effective in MDR- *A. baumannii* strains. Follow-up experiments affirmed its bactericidal and low-cytotoxic effect on human cells and showed how AI could be utilized to create pathogen-targeting antibiotics [40].

#### 3.3.3. Curcuminoid Derivatives: AI-Guided Optimization

An integrated QSAR and molecular docking model that was trained using an antibacterial and pharmacokinetic dataset was used to explore curcuminoid derivatives. The AI models were used to rank thousands of curcuminoid-like molecules depending on the predicted binding affinity with the outer membrane proteins of *A. baumannii*, membrane permeability, and ADMET. High-ranking compounds were prepared and tested in vitro, where they showed significant inhibitory activity and synergistic activity with traditional antibiotics. Time-kill kinetics and MIC experiments verified the increased bactericidal activity, which show how AI can guide the repurposing and optimization of natural-product-derived scaffolds [36].

#### 3.3.4. AMPs: AI-Guided Design

The trained DL models, including AMPs-Net were trained on the curated peptide databases that comprised thousands of known antimicrobial and non-antimicrobial sequences. These models acquired complicated sequence activity patterns, such as charge distribution, hydrophobicity, and motif structure. Generative models then generated new peptide designs that were optimized to have antimicrobial activity and less host toxicity. These peptides were confirmed by in vitro membrane disruption assays and bacterial growth inhibition experiments, where some of the AI-produced peptides had comparable or even superior activity compared to natural AMPs, which justifies the strength of AI in searching peptide sequence space [41].

#### 3.3.5. Phage Lysins: AI-Guided Discovery

Bacteriophage genomic data have been used in AI models to find lysins, which are enzymes that cause the breakdown of bacterial cell walls. ML classifiers were trained in order to identify sequence patterns related to lytic activity. The models have filtered the genomes of thousands of phages, which predict novel lysins with broad-spectrum activity against Gram-positive pathogens. Experimental validation inveterate strong antibacterial effects, prioritizing how AI can mine genomic “dark matter” for entirely new antibacterial modalities [42].

These case studies, taken in concert, indicate that AI-based pipelines are capable of quickly screening and pinpointing any chemically diverse antibiotic that has experimentally proven activity against MDR pathogens. Combining virtual screening and predictive modeling with laboratory testing, AI addresses the main weaknesses of traditional discovery and offers a solid translational route to the creation of the next-generation antibacterial agents.

## 4. AI in AMPs Discovery and Design

### 4.1. Relevance and Mode of Action of AMPs as Therapeutics

AMPs represent one of the largest categories of broad-spectrum antimicrobial agents with low tendency to cause AMR. Their mechanisms of action are to interfere with bacterial membranes, regulate immune responses, and attack intracellular elements. The antimicrobial activity and host toxicity of an AMP is dictated by a complex interrelation among physicochemical and structural properties, including net charge, hydrophobicity, and sequence motifs, that control its antimicrobial activity and host toxicity [43].

Nevertheless, AMPs have not been able to translate into drugs despite their potential due to the major challenges, namely, the high-throughput experimental screening of large peptide libraries, incomplete definition of structure–activity relationships, and the inability to optimize between potent antimicrobial activity and host biocompatibility. These bottlenecks have been historically had a slowing effect on the integration of novel AMPs into pharmaceutical pipelines [44]. However, these challenges make AMPs ideal targets for AI-driven discovery and optimization strategies.

### 4.2. Combined AI Systems of AMP Discovery and De Novo Design

To eliminate these obstacles, AI has already been used on two complementary, and increasingly unified, stages of the development chain: the discriminative identification of potential peptide candidates and the generative creation of new sequences.

**Phase 1:** Discriminative AI of Discovery and Prioritization. This is the first stage, at which ML systems, such as the current DL systems as AMPs-Net are trained as classifiers to recognize large datasets of known AMP and non-AMP sequences. These discriminative models are trained to recognize the pattern of complex features of antimicrobial activity and low toxicity. They screen genomic, metagenomic, or synthetic libraries at a very high rate to filter through millions of sequences to generate a shortlist of high-probability candidate peptide sequences to be experimentally validated, and in the process increase the aforementioned discovery rate several-fold [33,45].

**Phase 2:** Rational Design and Optimization Generative AI. Through this capability, generative AI models, including variational autoencoders (VAEs) and generative adversarial networks (GANs), are proactively designed. These models are trained using the same underlying information, and they learn the latent grammar of functional AMPs. Then they can do de novo peptides that are designed to have target properties, e.g., improved broad-spectrum activity, mechanism of action, and reduced cytotoxicity, to the extent of going off-target with non-natural amino acid sequence combinations [46].

**Synthesis and validation:** Bridging the gap with Computation and Experiment. The greatest uses of these AI paradigms are when the two are used in a closed-loop pipeline. Discriminative classifiers can immediately pre-screen novel seeds of generative model on desired attributes. Next, computational biophysics tools, especially MD simulations, can give atomistic information on the stability of these peptides and their binding to target membranes, which can be a valuable step of in silico validation prior to synthesis [47].

By efficiently discovering new systems, this iterative routine of generative design, discriminative AI, and physics-based simulations produces a very efficient discovery engine. The last and the most crucial step is still strong in vitro and in vivo experimental validation to ensure the biological activity and safety. Encouragingly, this combined AI-based system has already produced promising multimodal peptide candidates with attractive antimicrobial characteristics against pathogenic resistant microbes [48], providing a transformative future perspective in antimicrobial development.

## 5. Understanding AMR and Mechanism Prediction

### 5.1. AI for Predicting Resistance Mechanisms

Use of AI models has grown significantly over time to explain AMR mechanisms with the help of genomic, transcriptomic, and proteomic data. These methods help in figuring out the genetic determinants and the pathways that give resistance thus allowing the very first detection and the monitoring of the resistant strains that are newly arising [49].

**Figure 3:**
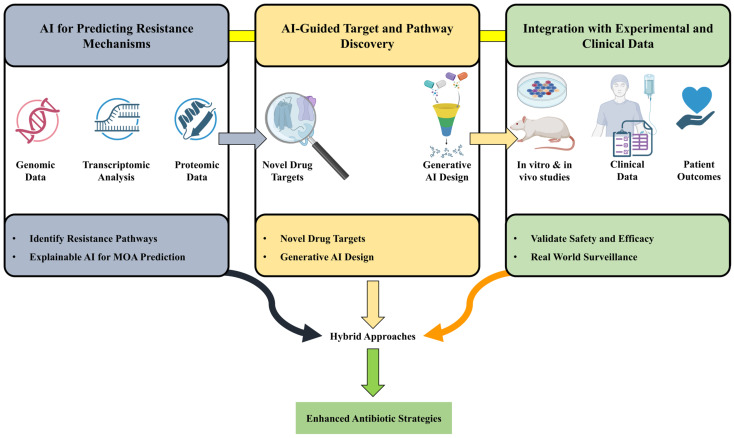
AMR and prediction of mechanism with the help of AI.

XAI techniques have become popular to lift up the interpretability level of the prediction of antibiotic mechanism-of-action (MOA). A good example is hierarchical classifiers that study transcriptome changes and in doing so are able to accurately identify primary MOA of the compounds which are already known and novel ones thus facilitating rational antibiotic development [50]. Furthermore, AI models that combine clinical and molecular data can lead to resistance evolution thus offering the most valuable insights for implementing therapeutic strategies in advance (Figure 3).

### 5.2. AI-Guided Identification of Novel Targets and Pathways

With the help of AI, the scientists can locate new druggable targets in the bacterial superbugs that cause serious infections and are resistant to drugs, by AI examines the most vital steps in the bacterial metabolism and the bacterial parts that are most susceptible to damage. Multi-target AI models created for multi-protein and multi-strain inhibition interact with resistance phenotypes’ complexity and, at the same time, help in the design of the versatile antibiotics which are able to overcome MDR [51]. Generative AI structures are set to molecular designs to foil the already known resistance mechanisms by changing the structural aspects that are responsible for the efflux or the enzymatic degradation (Figure 3).

### 5.3. Integration with Experimental and Clinical Data

For clinical relevance, it is essential that such initiatives are complemented by experimental validation. In contrast, AI-assisted analysis of in vitro assays such as MIC determination and time-kill studies, and in vivo models result in an increase in insights in efficacy and safety of compounds.

Both the accuracy and capability of the model can be refined by incorporating real-world clinical data, such as patient outcomes and resistance patterns. Hybrid approaches, combining AI with more traditional experimental methods, overcome these various limitations associated with biases in the models and predictive errors to enable the translation of AI innovations into therapeutic realities [52]. In addition to this, AI can process big data relating to health care-associated infections at much faster rates, hence making it a very robust tool for effective surveillance and stewardship practices (Figure 3).

## 6. Open Science, Data Sharing, and Collaborative Efforts

### 6.1. Importance of Accessibility and Collaborative Platforms

Huge quantities of accessible quality data are needed for the development of AI models in antibiotic discovery. Sharing the results of the screenings, genomic data, and phenotypic profiles openly is the only way to create AI algorithms that can be robust and generalizable to a wide variety of situations. It requires a team composition of computational scientists, microbiologists, chemists, and clinicians that support the creation of new ideas and are prepared to perform biological validation of the correctness of models used by AI.

Other approaches to dealing with difficulties arising from differences in data and quality control issues that might be responsible for poor model performance include defining standard protocols for data curation and sharing. The advantage of such measures is that it continuously eases dataset updates, thus making reproducibility easier and opening doors wider for community involvement that is indispensable for a fast development process [53].

### 6.2. Open-Source AI Tools and Benchmark Datasets

The community challenges driven by benchmarks motivate reproducibility and comparability, allowing the setting of baselines in performance and the discovery of best practices. Other successful fusions of AI pipelines’ goals with the acceleration of identification and optimization in antibiotic development are MoleculeX and SyntheMol. Wider general adoption of these tools democratizes access and fosters openness within AI-assisted drug discovery [8].

### 6.3. Challenges in Data Ethics, Privacy, and Algorithmic Bias

Different sources of data, especially when it comes to data provided by patients themselves, pose particular ethics that cannot be overemphasized when it comes to data control. Lack of proper data control will lead to data breaches or misuse, thereby undermining public trust and values in AI.

Bias in algorithms arising from non-representative datasets might pose threats to the fairness and generalizability of models, which might result in the exacerbation of healthcare inequities [54]. Some of the proposed approaches to address these challenges to allow safe usage of AI in antibiotic development, including those offered in drug development, are a transparent development process, working within strong ethical guidelines, and using an explainability method. Furthermore, the ethical use of AI necessitates unceasing supervision and adjustment in harmony with changes in regulatory standards and societal expectations [55].

These ethical and regulatory questions prove to be even more acute in the particular context of AI-discovered antibiotics, as low- and middle-income nations have the highest rates of AMR but have fewer digital and laboratory facilities. The models trained on data with a preponderance of data collected in high-income countries might not reflect the diversity of pathogens, resistance mechanisms and treatment practices in a particular region, resulting in biased or suboptimal results when implemented in a resource-constrained healthcare system. In addition, AI-designed drugs, which are expensive to manufacture, proprietary AI platforms, and intellectual property can limit access to life-saving antibiotics in settings that need them most.

Regulatively speaking, the agencies will need to consider not only the safety and efficacy of AI-discovered antibiotics but also the clarity, reproducibility, and traceability of the algorithms involved in the designing of the same. Insufficient regulatory guidelines to substantiate AI-generated drug candidates, especially those that are optimized through black-box DL models, creates uncertainty for clinical approval and global deployment. To overcome these obstacles, the following solutions will be necessary: international data-sharing standards, open and auditable AI models, and regulatory approaches that will guarantee innovation as well as fair access to AI-enabled antimicrobial therapies [56].

## 7. Challenges and Limitations of AI in Antibiotic Discovery

### 7.1. Technical and Methodological Barriers

Handling highly imbalanced and noisy datasets is just one of the biggest technical problems in AI-driven antibiotic discovery. In these datasets, for example, active compounds or resistant variants are very few. This kind of imbalance makes it very difficult to train accurate predictive models and therefore, it requires advanced algorithmic strategies and data augmentation techniques to solve the problem.

The interpretability of AI models is still very limited, as DL architectures used are generally a “black box” and hence, the trust of users and acceptance by regulatory authorities are affected [57]. The increased biological complexity and multigenic nature of AMR issues that are even more challenging when modeling hence require integrative and multi-scale approaches which need a lot of computational power [58]. The fact that AI decision-making processes are not transparent makes it hard to validate and clinically translate; thus, there is still a strong need for XAI frameworks.

These limitations have already shown distortions that are measurable through the practical antibiotic discovery projects. Numerous AI models are trained using public datasets, which are biased towards well-known chemical scaffolds and Gram-positive bacteria. Consequently, AI models at early stages often overpredicted the activity against Gram-negative pathogens, such as *A. baumannii*, due to the underrepresentation of compounds having to traverse the intricate outer membrane of the pathogen in the training data. Such bias has seen several instances where AI-prioritized molecules exhibited strong computational scores and failed to work in experimental validation because of poor permeability or efflux vulnerability. Similarly, the screening systems based on DL, are able to identify potent antibacterial hits successfully, but their black-box nature resulted in only limited information on mechanism of action, which makes it challenging to predict resistance as well as rationalize optimization. During the discovery of AMPs, AI models frequently produce peptides with high projected activity and high cytotoxicity or low serum stability, which is indicative of poor-quality annotations of toxicity and pharmacokinetics in most training datasets. These examples indicate that the bias of datasets and their low interpretability are not just a matter of theoretical vulnerability, but a direct implication on the reliability and translational usefulness of AI-based antibiotic discovery pipelines [33,59].

### 7.2. Translational and Clinical Challenges

It is not easy for experimental methods to validate the predictions made by computational methods. To confirm their effectiveness, toxicity, and pharmacological properties, AI-driven candidates usually need a lot of different tests both in vitro and in vivo. The process, which is very demanding in terms of resources, together with the requirements set by the regulatory bodies, are the main reasons why the progress is slow.

Assessment of safety and compliance with the regulatory standards are still the main obstacles that must be overcome before AI-discovered antibiotics can be used [60]. The use of AI techniques in the pharmaceutical development pipeline, which is already well-established, requires the change of workflows, validation protocols, and quality control measures in order to be able to receive new computational results. Moreover, the use of AI-derived therapies in the clinic will be possible only if there is solid evidence of their superiority or at least equivalence to the existing treatments [49]. These translational limitations highlight the importance of conjoining predictions with rigorous experimental and regulatory evaluation.

### 7.3. Socioeconomic and Infrastructure Constraints

Innovations in AI-powered antibiotic discovery require an adequate setup, powerful machines and labor force with proper skills [61]. Such condition is rarely found in low- and middle-income countries that suffer heavily from AMR. These differences in conditions could lead to the division in access to new therapies and diagnostic tools.

Investing in the promotion of local infrastructures, education and capacity building is a must if we want to see the positive impact of AI on a global scale in the fight against infectious diseases [62]. In addition, equal opportunities for technology transfer and data sharing must be created to avoid the impact of socioeconomic and geographical barriers.

### 7.4. Unanswered Questions and Critical Knowledge Gaps

Although the technologies are advancing at an incredible pace, there are a number of underlying questions and knowledge gaps, which need to be addressed to achieve all the potential of AI in the field of antibiotic discovery [63].

**The Explainability Gap in Mechanism Prediction:** AI models can be very accurate in predicting the antibacterial activity of a compound, but can we explain why it works the way it does? I.e., what is the structure of the mechanism of action (MOA) of that molecule? Black-box models are able to detect hits but cannot be used to give the mechanistic insight required to achieve rational optimality and to predict resistance. To fill this gap, the framework of XAI in biological systems needs to be developed.

**The Data Scarcity vs. Chemical Novelty Paradox:** AI models are data-intensive, but the most valuable discoveries are novel chemotypes that do not have close analogs in training datasets. One outstanding question whose answer remains unclear is how to construct models which can make reliable extrapolations into genuinely novel chemical space, as well as interpolations within familiar space. Other techniques, such as few-shot learning, meta-learning, and physics-informed neural networks are under investigation but not strong in this area.

**Combination of Multi-Scale and Dynamic Data:** The existing models are based mostly on fixed chemical structures and endpoint biological assays. A significant realization is the effective incorporation of dynamic, time resolved data—e.g., dynamic transcriptomic responses (e.g., ATAC-seq), dynamic membrane perturbation measurements, or host immune modulation dynamics—into predictive models. Drug–pathogen–host interaction dynamics are important to predict efficacy and toxicity in complex physiological conditions.

**Resistance Evolution vs. Resistance Presence:** The majority of models predict the occurrence of a current strain being resistant according to genomic markers. A much more complicated, and largely unresolved, objective is forecasting the path and probability of de novo resistance throughout the development of a new antibiotic. Designing evolution-proof therapies requires AI models capable of in vivo simulation of population dynamics and evolutionary pressure.

**The Translational Chasm to AI-Designed Peptides (AMPs):** There is an apparent and ongoing divide between the perceived efficacy of AMPs in a given system (e.g., positive predicted measures) and their efficacy in vivo. Top questions that remain unsolved include the ability to predict serum stability, tissue distribution, immunogenicity and metabolic clearance with just sequence data or structural data alone. These complicated pharmacological properties of peptides have less codified rules compared to small molecules and thus AI prediction is very challenging.

**Multi-Target and Synergistic Strategies:** AI has potential to be used to identify polypharmacological agents or synergies between drugs. Nevertheless, there are no well-developed experimental and computational models to test these multi-target hypotheses- and even to differentiate between a real synergetic effect and an additive one. The absence of quality, standardized combination screening data is a significant bottleneck.

## 8. Emerging Trends and Future Directions

### 8.1. Advances in Multi-Modal and Multi-Objective AI Models

New AI models have been progressively combining multi-omics datasets (chemical, genomic, transcriptomic, phenotypic) in order to fundamentally comprehend the problems of antibiotic discovery. These multi-modal approaches make it possible to create comprehensive models of drug–target interactions, resistance mechanisms, and efficacy profiles.

The multi-objective optimization computer algorithms can also consider, simultaneously, antibacterial efficacy, toxicity, pharmacokinetic properties, and resistance prevention in order to provide complex designs of antibacterial candidates [64]. There are applications of perturbation theory ML as well as efficient multi-task learning techniques being utilized to tackle complex biological networks in relation to heterogeneities in diseases as well as multi-strain bacterial infections. These strategies specifically focus on bridging the integration gap of multi-scale data in attempts to develop coherent models that are correlated with chemical input with dynamic phenotypic and omics outputs.

### 8.2. Integration of AI with Synthetic Biology and Nanotechnology

AI-assisted designs enable improving the properties of the agents such that the agents could have maximum specificity for the target, the agent’s shelf life could be extended, along with maximum safety being ensured with respect to the host [65].

In short, phage lysins are defined as the enzymes produced by bacteriophages, which break down the cell wall of bacteria and hence are a newly discovered, very intriguing source of non-conventional antibiotics. AI-based technologies are able to detect lysins by examining genomic dark matter and hence significantly lower the time span needed for the identification and study of the subject matter [66]. Moreover, the combination of computer modeling and automatic wet lab-based synthesis makes the development of novel medicines exponentially quicker. The synergy is aimed at bridging the translational gap in novel modalities (such as engineered phages or nano-formulations) by optimizing the delivery and production system of the therapeutic agent, but in addition, by optimizing the therapeutic agent itself (using AI).

### 8.3. Personalized Antibiotic Therapeutics and Predictive Stewardship

Person-specific models of AI represent the future for personalized antibiotics, ensuring that it becomes possible to select the optimal treatment in relation to the micro-flora. The use of AI in AMR monitoring in real time remains an immense help in terms of informed decisions related to optimized treatment. These kinds of adaptive models are able to swiftly react to newly emerging resistance trends, thereby modifying therapeutic recommendations at the clinical level so as to decrease resistance spread. The incorporation of predictive AI tools in healthcare facilities is a very good idea for the future as it can lead to better patient outcomes and the antibiotic existing can be used for a longer period of time. The final complexity of this direction is breaking the barrier of population-level predictions and patient-specific models [67]. One of the most important questions not yet properly answered in this regard is what will be the minimum, most clinically viable dataset (e.g., a quick microbiome read, host biomarker profile), in which AI can be reliably used to provide personalized advice.

### 8.4. AI in Antibiotic Development and Repurposing

Besides personalized therapeutics, AI is also being used to help hasten the creation of new antibiotics and find new applications of already-existing drugs. AI-driven can be used to design clinical trials in an optimal manner through predicting patient effects, stratification, and optimal dose and endpoints [68,69]. As an example, IBM Watson for Drug Discovery has been applied to clinical and molecular data to find subgroups of patients that might better respond to a certain antibiotic treatment [70]. In the same way, some AI-based simulations have been used to optimize trial procedures of novel antibiotics, saving time, money, and increasing the chance of clinical success.

AI is also crucial in the reuse of old antibiotics in the fight against resistant bacterial strains. ML algorithms are capable of processing large amounts of chemical, genomic and clinical data to discover previously unknown therapeutic opportunities. As an example, AI-driven research has proposed the use of the anti-parasitic medication, niclosamide, as an antibacterial agent against MDR Gram-negative bacteria [71]. Synergistic antibiotic combinations to boost the effectiveness of colistin, including meropenem, have also been predicted by DL models [72].

These solutions would not only reduce the time taken to develop antibiotics but also supply novel solutions to address AMR through combining AI with clinical and genomic data. The future of drug discovery and clinical implementation both as far as computational predictions and experimental validation is concerned have a promising outline.

## 9. Case Studies of AI-Driven Antibiotic Discovery

### 9.1. Natural Product Exploration and Streptomyces-Derived Drugs

One of the major sources of antibiotics in the past has been the *Streptomyces* species, among which Streptomycin and a number of other compounds used in the clinic are the most well-known examples. A conventional natural product screening has been faced with problems such as repetitive rediscovery and inefficiency; thus, there has not been much progress lately. AI-enabled genome mining of *Streptomyces* sp. shows to the world that there are a lot of biosynthetic gene clusters that can encode the so-called secondary metabolites which in turn can have antimicrobial properties [73].

ML models that are incorporated into the natural product drug discovery pipeline help to predict the biological activities of the compounds and also make it easy to carry out the optimization of the biosynthetic pathways. These measures go beyond the limitations of the traditional bottlenecks as they mainly deal with genome-guided discovery and computational prioritization. Nevertheless, most of the reported biosynthetic gene clusters encode poorly active metabolites, or they just recapitulate previously known metabolites, which is a significant shortcoming of AI methods using genome-mining.

### 9.2. AMP Discovery Pipelines

Novel peptide motifs to which enhanced antimicrobial and antiviral activities were associated have been identified by DL-based AMP discovery pipelines in a successful manner. The integration of computational prediction models with molecular dynamics simulations makes it possible to evaluate the stability of the peptide and its interaction with the microbial membrane; thus, the reliability of the selection is increased.

Experimental validation serves as a witness to the therapeutic promise of some AI-generated peptides, which show multifunctional antimicrobial effects [32]. Successes such as these form a compelling case for the efficacy of AI pipelines in tackling the issue of combinatorial complexity that is inherently evident when considering AMP discovery and optimization. Other discriminatory models, like Support Vector Machines, when combined effectively with DL, may enhance such a categoric level of accuracy regarding putative AMPs. Practically, a significant percentage of AI-predicted peptides cannot be verified experimentally because of their instability, rapid proteolytic breakdown, or even cytotoxicity, which proves that computational predictability is usually overstated.

### 9.3. Targeting MDR A. baumannii

AI-assisted discovery tools have been used to derive innovative antibiotics like Abaucin and optimized curcuminoids in the battle against the infections of MDR *A. baumannii*. Computational screening combined with molecular docking, ADME evaluation, and microbiological assay validation is applied to find agents against a hard-to-treat pathogen, showing AI’s ability to quickly identify antimicrobial candidates.

First of all, these molecules have been designed to specifically target the outer membrane proteins that are not only sources of the bacterial virulence but also the resistance, thus providing new possibilities of therapeutic intervention. The experimental data show that such AI-designed antibiotics can indeed decrease the bacterial load and even get past the resistance mechanisms, which therefore implies a great potential for clinical use. Despite the significance of AI-guided programs such the Abaucin pipeline, most of the compounds predicted by such pipelines fail to work in different clinical isolates or in vivo models, indicating overfitting to small laboratory datasets and missing modeling of bacterial physiology.

### 9.4. Lessons from Successes and Failures

Combined with the case studies, it can be concluded that although AI can dramatically speed up the process of identifying candidates, it cannot substitute experimental validation, medicinal chemistry, or clinical microbiology. The disconnect between computation prediction and biologic reality is one of the most and cannot be overcome by those derived by AI and not all AI-derived hits are survivable through subsequent testing.

## 10. Conclusions and Recommendations

### 10.1. Summary of AI’s Transformative Role in Antibiotic Discovery

AI has changed everything in the field of antibiotic discovery. With the help of AI tools, a lot of the work that was before done manually or by trial and error can now be done faster and with higher precision. AI is capable of going through a lot of chemical and biological data, quickly and accurately identifying molecular properties, and also creating totally new molecules by itself in order to fight AMR and to completely solve the problem of one of the major bottlenecks in anti-infective therapy.

This revolution is also being felt in the use of indigenous peptides, natural products, and small molecules in the discovery pipeline thus expanding the scope of therapeutics and at the same time facilitating the accurate targeting of pathogens resistant to drugs. Methods driven by AI have led to the fast implementation of drug development projects and give a possibility of continued innovation in antibiotic research.

The introduction of AI into the field of antibiotic discovery has been a game-changer, the whole process of discovery has been accelerated and made more efficient [74]. AI technologies such as ML, DL, and generative modeling can lead to the discovery of novel antimicrobial agents in a much shorter time, mechanisms of action can be predicted at a much faster pace, and optimization of both pharmacokinetic and toxicity parameters can be done at the same time. The technology links together the heterogeneous data from genomic and chemical libraries, and phenotypic screening output, and thus reveals the patterns and associations which were previously not accessible by conventional methodologies. AI-powered systems also enable higher throughput in virtual screenings, structure–activity relationship optimization, and design de novo small molecules and peptides with a higher level of potency and selectivity. There are examples like the discovery of the antibiotics Halicin and Abaucin in which AI was instrumental in finding completely novel antibiotic scaffolds that are effective against MDR pathogens. Furthermore, the combination of AI with multi-omics data, synthetic biology, and automated experimentation has not only shortened the time of discovery but it has also reduced the costs of development. These advances underscore not only existing achievements but also the potential for future AI-driven innovations in antibacterial drug discovery.

### 10.2. Addressing Current Challenges to Maximize AI’s Impact

The prospects of AI in antibiotic discovery can be transformative, yet, to be translated into clinical innovation, technical, biological, and ethical issues should be handled with caution. Availability of high-quality, standardized, and well-annotated datasets is the most important requirement. Experimental variability, inconsistent reporting of antimicrobial activity and paucity of toxicity and resistance data impact considerably on model robustness and reproducibility. Making established benchmark datasets and standardized experimental conditions should thus be developed to ensure credible model training and validation.

The second significant challenge is restricted generalizability of the existing AI models. The majority of learning algorithms are trained on rather small chemical and biological spaces resulting in overfitting and worse predictive performance when extended to new scaffolds or new bacterial strains. The issue is especially pronounced in the area of antibiotic discovery, as truly innovative chemotypes are not common in current datasets. To resolve this problem, a combination of transfer learning, few-shot learning, and physics-informed modeling methods will be needed that can make predictions in areas that have not been studied regarding chemical space.

The interpretability of models is also a significant impediment to practicality. DL systems can be considered black boxes, with minimal understanding of why a specific compound is predicted to be active or toxic. This reduces the scientific knowledge and makes prioritization of experiments difficult, as well as makes regulatory approval challenging. The use of XAI frameworks is thus paramount to connect molecular features and biological processes, allowing rational lead optimization and a better trust in the AI-generated predictions.

Moreover, the differences between the computational predictions and experimental outcomes are still prevalent. A lot of AI-identified hits do not succeed in biological validation due to the fact that the existing models do not accurately model complex biological processes like host–pathogen interactions, biofilm formation, immune modulation and metabolic stability. In addition, the low stereochemical representation and simplified molecular descriptors may also result in false positives and erroneous activity predictions. To bridge this gap, closer collaboration between AI and molecular dynamics, systems biology, and high-content phenotypic screening will be required.

Lastly, to make AI responsible and fair to use in antibiotic discoveries, ethical, economic, and infrastructural limitations should be addressed. The costs of high computation, lack of accessibility of data and computing resources, bias in the algorithms, and uncertainty about the ownership of the data can increase global inequalities in access to next-generation antibiotics. To make sure that the AI-driven antimicrobial discovery will help in both high- and low-resource environments, it will be necessary to create transparent systems of governance, open data projects, and international partnerships. Addressing these limitations proactively, through interdisciplinary collaboration, XAI, standardized datasets, and equitable access will ensure that AI fulfills its potential in antibiotic discovery.

### 10.3. Future Perspectives and Call for Multidisciplinary Integration

Further innovation in antibiotic discovery will be linked to the smooth fusion of AI with biotechnologies, including synthetic biology, genomics, nanotechnology, and high-throughput experimental platforms. To achieve this potential, researchers need to focus on interdisciplinary teams, comprising computational scientists, chemists, microbiologists, and clinicians, to create explainable models of AI, experimentally test predictions, and combine multi-omics with clinical data in a manner that creates more accurate and biologically relevant predictions [75]. Funding agencies play a vital role in facilitating infrastructure, standardized and open-access datasets, and translational research tools that facilitate the transfer of the computational predictions into clinical use, and promote international and cross-institutional collaboration to speed up innovation. In their turn, policymakers should develop regulatory systems and ethical standards to guarantee the responsible and fair uses of AI-based antibiotic discovery, such as open data management, algorithmic equity, and general accessibility in both high- and low-resource contexts. Through the creation of a coordinated action in these areas, the creation of AI-assisted antimicrobial discovery may become a globally collaborative, data-driven initiative that can effectively respond to the growing menace of AMR.

## Figures and Tables

**Table 1:** AI Tools and platforms for antibiotic discovery.

Tool	AI Approach Used	Primary Application	Data Sources	Notable Outcomes
DeepChem	ML/DL library (TensorFlow-based)	Molecular property prediction, virtual screening	ChEMBL, PubChem, custom SMILES datasets	Used for antibiotic QSAR modeling and toxicity prediction
Chemprop	Graph Neural Networks (GNNs)	Predicting bioactivity and ADMET profiles	Molecular graphs from ZINC15/ChEMBL	Outperformed random forest models in activity prediction
GNINA	CNN-based docking	Protein–ligand scoring and pose prediction	Protein Data Bank (PDB)	Improved binding pose ranking accuracy for antibiotic targets
AMPs-Net	Deep Neural Network (CNN + RNN hybrid)	AMPs classification	AMP benchmark datasets	High accuracy in identifying novel peptide motifs
SyntheMol	Reinforcement Learning + Monte Carlo Tree Search	Generative small-molecule design	Graph-based compound library	Produced multi-objective antibiotic candidates
MoleculeX	Generative Transformer + GNN	De novo drug design and optimization	ChemBL, BindingDB	Created new scaffolds with predicted antibacterial activity
BioGPT/ChemBERTa	Large Language Models (LLMs)	Literature mining, retrosynthetic prediction	PubMed, patents, chemical text	Automates hypothesis generation and reaction prediction
AlphaFold2/RoseTTAFold	DL for protein folding	Target structure prediction	Genomic and proteomic databases	Enabled structure-based antibiotic target modeling

**Table 2:** AI-driven antibiotic discovery pipeline from data to clinic.

Stage	Input Data	AI/Bioinformatics Tools	Output	Role in Drug Discovery
Data generation	Genomes, chemical libraries, phenotypic assays	Sequencing, HTS, omics platforms	Raw biological and chemical data	Defines search space
Data preprocessing	Raw sequences, structures	AlphaFold2/3, ChEMBL, PDB, annotation pipelines	Structured protein, ligand, and activity data	Enables machine learning
Feature extraction	Molecules, peptides, targets	QSAR, molecular descriptors, embeddings	Numerical representations	Model input
Predictive modeling	Feature matrices	ML, DL, GNNs	Activity, toxicity, ADMET predictions	Candidate prioritization
Generative design	Trained models	VAEs, GANs, transformers	Novel molecules/peptides	Lead generation
In silico validation	Predicted candidates	Docking, MD, free energy models	Ranked and filtered hits	Reduces wet-lab burden
Preclinical optimization	Shortlisted hits	MIC, cytotoxicity, animal models	Biological efficacy	Confirms activity

**Table 3:** Case studies of AI-discovered or -optimized antibiotics.

Compound/Discovery	AI Technique Used	Target/Pathogen	Key Outcome	Reference/Source
Halicin	Deep neural network (DL-based activity prediction)	Broad-spectrum (esp. *A. baumannii*)	Identified novel scaffold with new MoA; validated in mice	Stokes et al., 2020 [39]
Abaucin	ML-guided virtual screening + molecular docking	*A. baumannii*	Narrow-spectrum, potent against MDR strains	Awan et al., 2024 [40]
Curcuminoid Derivatives	QSAR + molecular docking + ML ranking	*A. baumannii* OMP targets	Identified synergistic drug candidates	Boulammane et al., 2024 [36]
Synthetic AMPs (AMPs-Net)	Deep generative model	Gram-positive and Gram-negative bacteria	Generated peptides with enhanced selectivity and stability	Ruiz et al., 2022 [41]
Phage Lysins	AI mining of bacteriophage genomes	MDR Gram-positive pathogens	Predicted lytic enzymes with broad antibacterial activity	Zang et al., 2024 [42]

## Data Availability

No new data were created.

## Abbreviations

The following abbreviations are used in this manuscript:

AMR	Antimicrobial resistance
AI	Artificial intelligence
AMPs	Antimicrobial peptides
MDR	Multidrug-resistant
XRD	Extensively drug-resistant
ML	Machine learning
DL	Deep learning
NLP	Natural language processing
GNNs	Graph Neural Networks
PDB	Protein Data Bank
LLMs	Large Language Models
SMOTE	Synthetic Minority Over-Sampling Technique
GANs	Generative adversarial networks
VAEs	Variational autoencoders
QML	Quantum machine learning
QSAR	Quantitative Structure–Activity Relationship
ADME	Absorption, Distribution, Metabolism, and Excretion
MOA	Mechanism of action
